# Harnessing *Lactobacillus*-derived SCFAs for food and health: Pathways, genes, and functional implications

**DOI:** 10.1016/j.crmicr.2025.100496

**Published:** 2025-10-20

**Authors:** Yousef Nami, Milad Shaghaghi Ranjbar, Mahmoudreza Modarres Aval, Babak Haghshenas

**Affiliations:** a Department of Food Biotechnology, Tabriz Branch, Agricultural Biotechnology Research Institute of Iran (ABRII), Agricultural Research, Education and Extension Organization (AREEO), Tabriz, Iran; b Department of Microbiology, Faculty of Biological Sciences, Islamic Azad University, Kish International, Kish, Iran; c Regenerative Medicine Research Center (RMRC), Health Technology Institute, Kermanshah University of Medical Sciences, Kermanshah, Iran

**Keywords:** Lactobacillus, Short-chain fatty acids (SCFAs), Probiotics, Synthetic biology, Gut microbiome

## Abstract

Short-chain fatty acids (SCFAs) are crucial microbial metabolites that mediate host–microbiota interactions, regulate immune responses, and maintain gut homeostasis. While most studies focus on SCFA production by obligate anaerobes, recent evidence highlights *Lactobacillus* spp. as potential SCFA-producing microorganisms that remain understudied in terms of SCFA-based health and food-related effects. This research review compiles what is currently known about species- and strain-specific biosynthetic capabilities of *Lactobacillus* for SCFA production, particularly acetate, and lactate, which, while produced by *Lactobacillus*, is not classified as a SCFA but plays similar metabolic roles (*e.g.*, activation of G-protein-coupled receptors, inhibition of histone deacetylases, and immune cell metabolism).

We additionally assess new synthetic biology and metabolic engineering approaches to improving SCFA yield in *Lactobacillus*, including gene circuit design, CRISPR editing, and co-culture optimization. Importantly, we discuss translational opportunities not only in inflammatory, metabolic, and neuroimmune diseases but also in the development of functional foods, synbiotics, and nutraceutical applications. Finally, we highlight key challenges—strain variability, delivery strategies, and regulatory oversight—that preclude both clinical and food system translation.

By integrating perspectives from microbiology, immunology, food science, and bioengineering, this review provides a broad framework for the rational design of SCFA-producing *Lactobacillus* strains as next-generation probiotics and food-grade biotherapeutics.

## Highlights

• *Lactobacillus* strains can produce SCFAs with immunoregulatory properties.
• Synthetic biology enhances SCFA yield and strain-specific immune modulation.
• Engineered probiotics offer new therapeutic potential in gut–immune disorders.

## Graphical abstract

**Figure:**
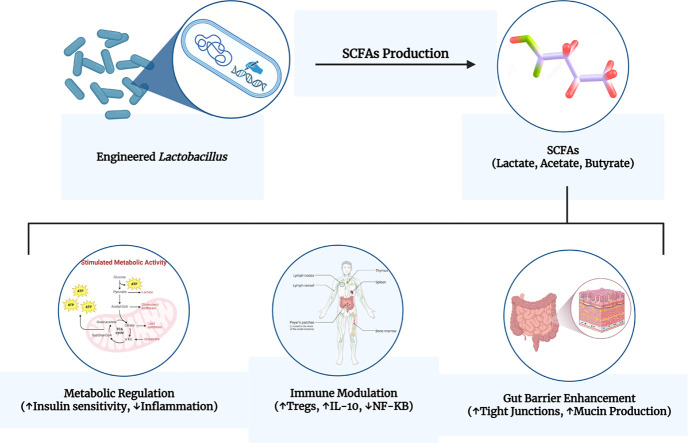

## Introduction

The gut microbiome is integral to host physiology, regulating immunity, energy metabolism, and mucosal homeostasis. Among its metabolites, lactate is produced in similar pathways but is not typically classified as an SCFA. The true SCFAs—acetate, propionate, and butyrate—remain key mediators of gut–immune crosstalk and barrier function, with growing evidence linking SCFAs profiles to systemic health and disease. Recent syntheses emphasize SCFAs as central effectors of diet–microbiome–host interactions (Zhang et al., 2023).

We highlight that *Lactobacillus*-derived SCFA production is generally modest compared to the concentrations needed for effective immunomodulation. Physiological concentrations of SCFAs required for immune modulation are typically in the range of 1–5 mM for acetate and 0.5–2 mM for butyrate (Tan et al., 2014). However, even the best *Lactobacillus* strains often produce SCFAs in the 10–20 mM range *in vitro*, which is much higher than what is typically achieved in the gut, where concentrations of SCFAs are diluted and can vary widely based on diet, microbiota composition, and local conditions (Ghosh et al., 2022). This highlights the challenge of scaling up the natural production of SCFAs for therapeutic applications and underscores the importance of bioengineering strategies to enhance SCFA yield and improve clinical relevance.

Historically, primary SCFA production in the colon has been attributed to obligate anaerobes such as Faecalibacterium prausnitzii and Roseburia spp. However, selected *Lactobacillus* (now distributed across multiple split genera within *Lactobacillaceae*) can also contribute to SCFAs pools—most consistently acetate and lactate—via strain-specific carbohydrate fermentation routes (*e.g.*, phosphoketolase and pyruvate-to-acetyl-CoA pathways) and, in consortia, by supporting cross-feeding toward butyrate (Dempsey and Corr, 2022; Zheng et al., 2020).

This strain-level diversity reflects the broader genomic and ecological heterogeneity of the former *Lactobacillus* genus after its 2020 taxonomic reclassification, which has reframed how metabolic capacity is mapped across species and strains relevant to human health (Oberg et al., 2022; Zheng et al., 2020). However, important gaps remain in our understanding of how *Lactobacillus*-derived SCFAs influence immune signaling and host-specific outcomes, underscoring the need for integrative microbiology–immunology approaches.

Beyond descriptive microbiology, the immunomodulatory actions of SCFAs depend on concentration, epithelial uptake, receptor engagement (*e.g.*, GPR41/FFAR3 and GPR43/FFAR2), and local microbial context—variables that shape outcomes in inflammatory and metabolic settings (Zhang et al., 2023). These actions also include inhibition of histone deacetylases (HDACs), an additional layer of epigenetic regulation. However, translational gaps remain between engineered metabolic outputs and measurable benefits in preclinical or clinical settings, which must be addressed before clinical deployment.

Concurrently, synthetic biology has unlocked tools to tune SCFAs outputs in *Lactobacillus* and related lactic acid bacteria (LAB), including CRISPR-based editing, promoter engineering, and pathway rewiring—moving these organisms from “naturally producing probiotics” toward “programmable therapeutic chassis” (Mu et al., 2022; Xie et al., 2024). In parallel, their natural role in traditional and modern fermented foods highlights the intersection of microbial metabolism with food science, where strain-specific SCFA production contributes not only to health but also to sensory and nutritional properties of food products.

We synthesize (i) strain-specific biosynthetic capacity for SCFAs production in *Lactobacillus* sensu lato, (ii) the immunological effects of these metabolites, and (iii) emerging engineering and delivery strategies to enhance SCFAs-mediated benefits (Dodero et al., 2024). This review connects quantitative metabolic outputs to immunological and translational relevance, providing a framework for rational probiotic and functional food design by linking microbial metabolism with host physiology.

### Literature search strategy

To improve transparency and reduce selection bias, we performed a structured literature search in PubMed, Scopus, and Web of Science. The following keywords and Boolean combinations were used: “*Lactobacillus*”, “short-chain fatty acids” OR “SCFAs”, “synthetic biology”, “bioengineering”, and “probiotics”. We considered publications from 2000 to 2025 written in English. Both original research and review articles were included if they directly addressed: (i) the role of *Lactobacillus* spp. in SCFAs biosynthesis, (ii) metabolic engineering or synthetic biology approaches for enhancing SCFAs production, or (iii) host immunometabolic responses linked to *Lactobacillus*-derived SCFAs. Studies not directly related to SCFAs mechanisms were excluded. Titles and abstracts were screened first, followed by full-text evaluation. While this review remains narrative in scope, these structured criteria enhance reproducibility and minimize bias.

### Overview of SCFAs: chemistry, sources, and core functions

SCFAs are saturated aliphatic monocarboxylic acids containing one to six carbon atoms: acetate (C_2_), propionate (C_3_), and butyrate (C_4_), and are the primary fermentation end-products of anaerobic carbohydrate metabolism in the gut and are essential for host–microbiota symbiosis (Den Besten et al., 2013). SCFAs are mainly produced in the colon at 50–150 mM concentrations, which vary considerably based on host diet, microbiota composition, and intestinal transit time (Ghosh et al., 2022).

SCFAs differ in solubility, pKa, and membrane permeability, influencing their biological activities. Acetate, for example, is the most abundant SCFA and is rapidly absorbed by the body and transferred to the peripheral circulation. At the same time, butyrate is readily taken up by colonocytes and acts primarily as an energy source for intestinal epithelial cells and as an HDAC inhibitor (Silva et al., 2020). These differences in physicochemical properties help explain the functional specificity of each SCFA in their roles in immune modulation, gut barrier maintenance, and energy metabolism.

Traditionally, only obligate anaerobes such as *Faecalibacterium prausnitzii* and *Roseburia* spp. were recognized as the leading SCFA producers in the gut (Louis and Flint, 2017). However, facultative anaerobes—including *Bifidobacterium* and *Lactobacillus* spp.—also produce SCFAs, with production capacity varying within species according to strain-specific metabolic pathways (*e.g.*, acetyl‑CoA conversion, pyruvate fermentation, Wood–Ljungdahl pathway), as demonstrated by strain-level differences in SCFAs output (Pessione et al., 2015; Thananimit et al., 2022). This metabolic versatility supports the application of *Lactobacillus* spp. as live biotherapeutic products (LBPs), particularly in gut niches where obligate anaerobes fail to establish stable colonization due to their lower tolerance to fluctuating oxygen levels and other environmental stressors (Brittain and Finbloom, 2025).

SCFAs influence host physiology in various ways. First, they can act as signaling molecules through G protein-coupled receptors (GPR41, GPR43, GPR109A), influence gene expression through epigenetic processes (HDAC inhibition), and maintain intestinal barrier function by enhancing tight junction formation and mucin synthesis (Tan et al., 2014). Second, SCFAs affect host immunity by promoting regulatory T cell (Treg) differentiation, inhibiting NF-κB activation, and modifying antigen-presenting cell (APC) function (Thorburn et al., 2014). As such, SCFAs act as central mediators connecting microbial metabolism with host immunometabolism, and their balance is closely tied to the maintenance of eubiosis or the onset of dysbiosis. This makes them critical targets for mechanistic studies and for developing translational interventions in metabolic, inflammatory, and neuroimmune disorders. In the following sections, we discuss specific roles of *Lactobacillus* spp. in SCFAs production and their immune-related effects (Parada Venegas et al., 2019; Silva et al., 2020).

### *Lactobacillus* and SCFAs biosynthesis: strain-specific capacities

*Lactobacillus* includes over 250 metabolically diverse species reclassified into several genera, such as *Lacticaseibacillus, Ligilactobacillus, and Latilactobacillus* (Zheng et al., 2020). These species colonize various ecological niches, most notably the human gut, where they are well known as probiotics, though not as primary SCFAs producers (Mazlumi et al., 2022; Shahverdi et al., 2023). Evidence shows that some specific strains of *Lactobacillus* spp. can produce relatively higher levels of acetate and lactate, as well as lower but physiologically relevant levels of butyrate and propionate. However, this production remains highly strain-dependent and often modest compared with canonical SCFA producers (Ríos-Covián et al., 2016). This variability underscores the need for metabolic engineering strategies to enhance SCFA output.

In *Lactobacillus*, SCFAs biosynthesis is highly related to central carbon metabolism, as most SCFAs are produced during carbohydrate fermentation as either homofermentation (Embden–Meyerhof–Parnas pathway) or heterofermentation (phosphoketolase pathway) (Marcobal and Sonnenburg, 2012). For example, heterofermenting species, such as L. *reuteri* and L. *fermentum*, catalyzing acetoclastic [acetyl-phosphate from acetyl-CoA to acetate], produce acetate as their primary SCFAs. Homofermenters like *Lactobacillus acidophilus* may produce SCFAs biomass catalyzed by lactate dehydrogenase to predominantly lactate, with negligible SCFAs products. Adding external electron acceptors (such as citrate, oxygen, or nitrate) can considerably impact SCFAs yield, specifically through modifications to the redox balance or energy conservation (Hatti-Kaul et al., 2018).

It is worth noting that particular *Lactobacillus* species can affect butyrate production indirectly through metabolic cross-feeding. For example, lactate and acetate produced by *Lactobacillus* can serve as substrates for butyrogenic bacteria like *Eubacterium hallii* and *Anaerobutyricum spp*., and the lactate and acetate can collectively enhance SCFAs production (Hatti-Kaul et al., 2018). In addition, the butyryl-CoA: acetate CoA-transferase–encoding gene has been engineered into *Lactobacillus* strains, enabling low-efficiency *de novo* butyric acid synthesis (Song et al., 2017).

**Fig. 1:**
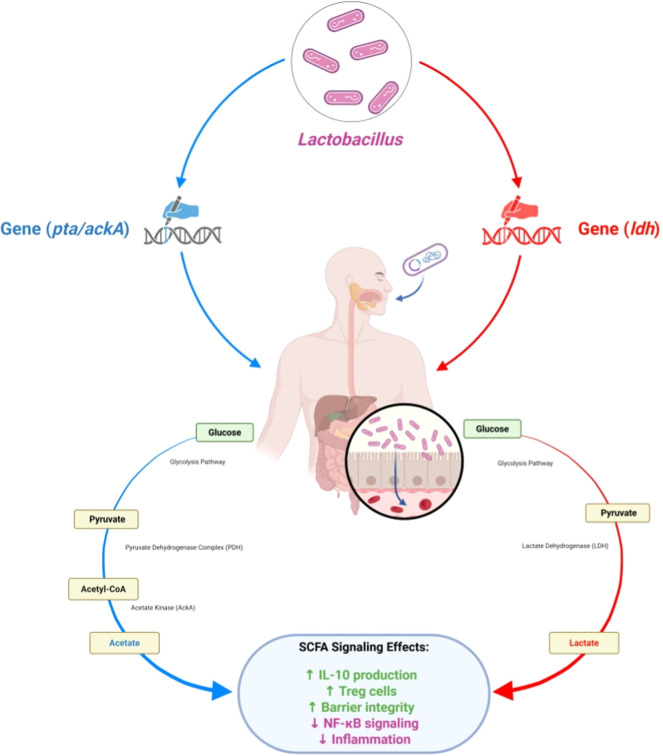
Schematic representation of SCFA biosynthesis pathways in Lactobacillus spp. Pathways are annotated with representative strains and relative flux contributions where data are available (e.g., ∼50 % increase in butyrate in F. prausnitzii/B. catenulatum co-culture vs mono-culture, (Kim, 2020 #45); total colon SCFA range ∼50–150 mM, (Wu et al., 2016).

Comparative genomic studies have identified key genes (*ackA, pta, ldh*, and *adhE*) that are present in some *Lactobacillus* strains but absent in others, explaining strain-specific differences in SCFAs biosynthesis (B. Wang et al., 2022). Recent multi-omics studies combining metabolomics and transcriptomics have revealed how *Lactobacillus* strains differentially regulate metabolic and gene expression profiles under gut-relevant conditions—insights that can be leveraged to enhance strain-specific SCFAs production for therapeutic applications (Huang et al., 2023; Thananimit et al., 2022). The main SCFA biosynthetic pathways and related gene clusters in *Lactobacillus* spp. are illustrated in Fig. 1. *Lactobacillus*'s SCFAs-producing capacity is strain- and context-specific, shaped by metabolic genotypes, substrate availability, and microbial consortia. Knowing and leveraging this variability will be essential for rational design of probiotics, particularly when targeting SCFAs-mediated immunometabolic pathways. Table 1 summarizes the major SCFAs produced by *Lactobacillus* spp., their main biosynthetic pathways, key genes involved, physiological roles, and representative referencesFig. 2.

**Table 1:** SCFAs production by Lactobacillus spp. – Representative strains, main pathways, key genes (★ core, ✦ accessory), reported yields, host effects, and notable features.

Strain (Species)	SCFAs Produced	Main Biosynthetic Pathway	Key Genes (★ Core / ✦ Accessory)	Reported yield (mmol/L or range)	Host Effect Evidence	Notable Features	References
L. plantarum 299v	Acetate, Lactate	Heterofermentative (phosphoketolase)	pta ★, ackA ★, ldh ★	Acetate ∼12–18 mmol/L (in vitro, glucose); Lactate ∼10–15 mmol/L	Reduced gut permeability, ↓ IL-6	Clinically studied; supports mucosal barrier	(Gänzle, 2015; Louis & Flint, 2017)
L. rhamnosus GG	Acetate, Lactate	Homofermentative (EMP pathway)	ldh ★	Acetate ∼8–10 mmol/L (mouse cecum); Lactate ∼5–7 mmol/L	↑ IL-10, Treg activation (observed in trials; effective SCFA concentrations typically ∼1–10 mM in vitro)	Widely used probiotic; immunomodulatory potential	(Koh et al., 2016; Ma et al., 2024)
L. reuteri DSM 17,938	Acetate, minor butyrate	Acetyl-CoA pathway + lactate cross-feeding	pta ★, ackA ★, buk ✦	Acetate ∼6–9 mmol/L; Butyrate <2 mmol/L (engineered strains)	↓ Inflammation in colitis models (low-level SCFA production may require cross-feeding for in vivo relevance)	Capable of low-level butyrate via engineered pathways	(Flint et al., 2024; Song et al., 2017)
L. paracasei F19	Acetate	Citrate-enhanced acetogenesis	citC ✦, pta ★, ackA ★	Serum acetate ↑ ∼5–7 mmol/L (human trial)	↑ Insulin sensitivity, ↓ adiposity (linked to modest increases in serum acetate; thresholds not fully established)	Linked to metabolic improvements in obesity	(Hatti-Kaul et al., 2018)
Engineered L. casei	Butyrate (heterologous)	CRISPR-mediated gene insertion	but ✦, buk ✦	Butyrate ∼4–6 mmol/L (in vitro, synthetic pathway)	Preclinical only	Synthetic strain with butyrate capability	(Song et al., 2017)
L. fermentum ME-3	Acetate, Lactate	Redox-modulated SCFA profile	ldh ★, pta ★	Acetate ∼7–10 mmol/L; Lactate ∼6–9 mmol/L	Antioxidant and anti-inflammatory effects (dual action via SCFA + glutathione; in vivo relevance still under study)	Dual action via SCFA and glutathione pathways	(Gänzle, 2015; Ma et al., 2024)
L. rhamnosus GR1	Acetate, Lactate	Homofermentative (EMP pathway)	ldh ★	Acetate ∼7–9 mmol/L (human trials); Lactate ∼4–6 mmol/L	↑ IL-10 production, modulates immune response (effective SCFA concentrations typically ∼1–5 mM in vivo)	Known for immunomodulatory effects	(Ma et al., 2024; Liu et al., 2023)
L. acidophilus NCFM	Acetate, Butyrate	Heterofermentative (phosphoketolase)	pta ★, ackA ★, buk ✦	Acetate ∼5–8 mmol/L; Butyrate ∼1–3 mmol/L	↑ Treg induction, ↓ pro-inflammatory cytokines (effects observed in gut models)	Probiotic with both anti-inflammatory and gut barrier properties	(Liu et al., 2023; Ma et al., 2024)

**Fig. 2:**
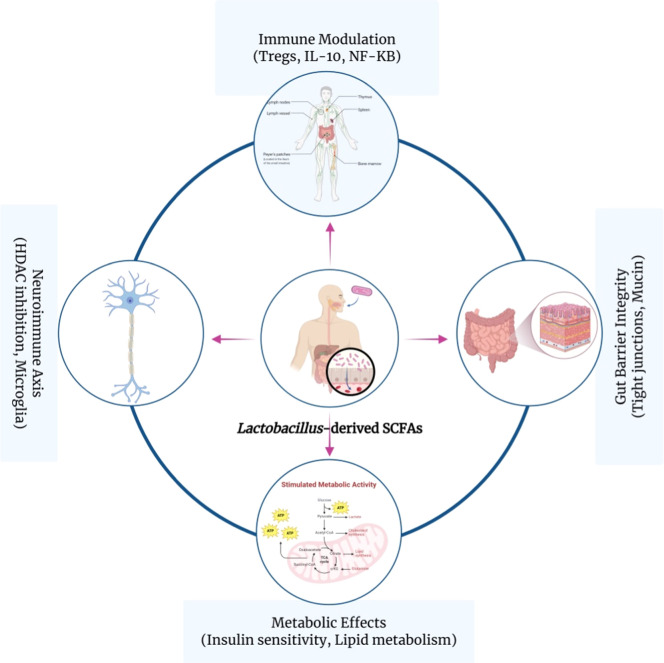
Conceptual overview of immunometabolic host effects of Lactobacillus-derived SCFAs.

### Pangenome of bacteria and lactobacilli: genetic diversity and SCFA-Relevant traits

The concept of the bacterial pangenome encompasses the total gene repertoire of a species, including the core genome (genes shared by all strains), the accessory genome (genes present in some but not all strains), and unique genes found in individual isolates. Pangenomic diversity is a key determinant of phenotypic variability, metabolic capacity, and ecological adaptability. In addition to chromosomal diversity, mobile genetic elements (MGEs)—such as plasmids, transposons, prophages, and integrative conjugative elements (ICEs)—play a crucial role in shaping bacterial genomes by facilitating horizontal gene transfer (HGT) and genome rearrangements (Haudiquet et al., 2022).

In pathogenic bacteria, pangenome plasticity often underlies antibiotic resistance and virulence factor acquisition, as observed in *Pseudomonas aeruginosa* and *Acinetobacter baumannii*, where transposable elements, integrons, and genomic islands contribute to rapid genetic adaptation (Karampatakis et al., 2024,^2025^). While probiotic *Lactobacillus* spp. are generally non-pathogenic, similar mechanisms of genomic plasticity contribute to their strain-specific metabolic versatility, including their ability to produce SCFAs.

In *Lactobacillus* and related genera, comparative pangenome studies have revealed substantial variability in carbohydrate utilization operons, stress response genes, and enzymes directly or indirectly linked to SCFAs biosynthesis (Sun et al., 2015). For example, genes encoding acetate kinase (*ackA*) and phosphotransacetylase (*pta*), central to acetate production, are present in most heterofermentative *Lactobacillus* species but absent in certain homofermenters. Plasmid-borne genes can provide additional metabolic pathways, including citrate utilization, which enhances acetate yield under specific growth conditions.

Moreover, *Lactobacillus* pangenomes often contain variable clusters for pyruvate metabolism, lactate dehydrogenase isoforms, and cross-feeding capacities that facilitate butyrate synthesis in consortia with butyrogenic bacteria (Goh et al., 2021; Sun et al., 2015). This genetic variability explains why SCFAs production capacity is highly strain-specific and influenced by both genomic content and environmental context. Pangenome analysis thus offers a valuable framework to identify metabolic determinants and mobile elements that can be harnessed in metabolic engineering strategies for enhanced SCFA yield. Integrating pangenomic data with functional assays will be critical for rational selection or design of *Lactobacillus* strains with optimized SCFA profiles for therapeutic applications. Table 2 summarizes the main pangenome features of *Lactobacillus* spp., highlighting the distribution of core and accessory genes, mobile genetic elements, and their relevance to SCFAs biosynthesis.

**Table 2:** Pangenome features of Lactobacillus spp. relevant to SCFA production – Core genes encode essential functions, while accessory genes and mobile elements provide strain-specific adaptations, including variable SCFA biosynthetic capacity.

Feature	Core Genome	Accessory Genome	Biological Significance	Examples	References
Genome size	∼1500–1800 genes	Variable	Determines essential functions and metabolic flexibility	L. plantarum, L. rhamnosus	(Wuyts et al., 2017)
Mobile genetic elements (MGEs)	Rare	Frequent	HGT, adaptation to niches	Plasmids, IS elements	(McInerney, 2023)
Antibiotic resistance genes	Rare	Occasional	Survival under stress conditions	ermB, tetW	(Sharma, Tomar, Sangwan, Goswami, & Singh, 2016)
SCFA biosynthesis genes	Stable	Variable in some pathways	Determines SCFA type and yield	pta, ackA, buk	(Goh & Barrangou, 2021)

### Toll-like receptors (TLRs) and their relevance to *lactobacillus*-derived SCFAs

Toll-like receptors (TLRs) are critical pattern recognition receptors (PRRs) that help detect microbial components and modulate innate immune responses. In the gut, TLRs are essential in maintaining immune homeostasis and defense mechanisms against pathogens. SCFAs, particularly acetate and butyrate, have been shown to modulate TLR signaling pathways, influencing immune responses and intestinal barrier function. Specifically, SCFAs can inhibit TLR4-mediated inflammatory responses by reducing NF-κB activation and pro-inflammatory cytokine secretion, contributing to gut health and immune modulation (Vinolo et al., 2011).

While TLR signaling plays a pivotal role in both protective immunity and inflammatory pathology, SCFAs produced by *Lactobacillus* spp. can regulate TLR activity to maintain immune balance. For example, acetate and butyrate have been shown to downregulate TLR4-mediated inflammatory responses, a key pathway involved in diseases such as inflammatory bowel disease (IBD). By modulating TLR signaling, SCFAs produced by *Lactobacillus* strains can help prevent excessive inflammation while promoting beneficial immune responses.

This section connects the role of SCFAs in immune modulation to TLR pathways, emphasizing how *Lactobacillus*-derived SCFAs influence gut-associated immune functions and highlighting their potential therapeutic implications for gut inflammation and other immuno-inflammatory conditions Table 3.

**Table 3:** Representative concentrations and immunological effects of Lactobacillus-derived short-chain fatty acids (SCFAs). The table summarizes experimental concentrations tested in vitro and in vivo, the cellular or animal models used, and the corresponding immune pathways or outcomes, highlighting their relevance to barrier integrity, anti-inflammatory responses, and Treg expansion.

SCFA	Concentration tested (mM/µM)	Model (in vitro / in vivo)	Immune pathway/outcome	Reference
Acetate	1–5 mM	Mouse T cells (in vitro)	↑ FOXP3, ↑ IL-10, Treg expansion	(Arpaia et al., 2013)
Butyrate	0.5–2 mM	DCs (in vitro)	HDAC inhibition, ↓ NF-κB activation, tolerogenic DCs	(Corrêa‐Oliveira et al., 2016)
Acetate	20–40 mM (colonic lumen); <1 mM (systemic)	Human colonic samples/plasma	Maintains barrier integrity	(Zhang et al., 2023)
Butyrate	1–3 mM	IECs	↑ Muc2 expression, enhanced barrier	(Furusawa et al., 2013)
Lactate	5–10 mM	IECs	↑ NLRP3 activation, antimicrobial peptide RegIIIγ	(Niu et al., 2023)
Propionate	0.5–1 mM	Macrophages	Anti-inflammatory phenotype (M2 polarization)	(Rodríguez et al., 2025)

### Immunomodulatory effects of SCFAs

SCFAs are well-established immunological modulators that influence host immune responses through multiple mechanisms. Although much of the current knowledge comes from studies on butyrate-producing *Clostridia*, increasing evidence supports the immunomodulatory roles of SCFAs, including acetate and lactate, produced by various microbiota, including *Lactobacillus* spp. Lactate is metabolically linked but not classified as a SCFA. However, these immunomodulatory effects are not unique to *Lactobacillus*-derived SCFAs but are characteristic of SCFAs in general. Thus, while *Lactobacillus*-derived SCFAs may contribute to immune modulation, their effects are consistent with the broader functions of SCFAs produced by other microbial species (Yongsen Wang et al., 2025).

SCFAs can mediate host cell interactions through receptor-mediated signaling and epigenetic mechanisms. The most described receptors for SCFAs are GPR41 (FFAR3), GPR43 (FFAR2), and GPR109A, which are highly expressed on intestinal epithelial cells (IECs), dendritic cells (DCs), neutrophils, and T lymphocytes (Du et al., 2024). SCFAs bind to these G-protein-coupled receptors (GPCRs), triggering anti-inflammatory responses, enhancing mucosal barrier function, and inhibiting the production of pro-inflammatory cytokines such as TNF-α, IL-6, and IL-1β (Ney et al., 2023).

In innate immunity, SCFAs, such as acetate and butyrate, regulate neutrophil chemotaxis and macrophage polarization. Acetate and propionate, through GPR43 signaling, dampen pro-inflammatory cytokine release (TNF-α, IL-6, IL-1β). Similarly, butyrate, at concentrations ranging from 0.5 to 2 mM, inhibits histone deacetylases (HDACs) in dendritic cells, limiting NF-κB activation and promoting tolerogenic phenotypes (Corrêa‐Oliveira et al., 2016). SCFAs also influence adaptive immunity by modulating T cell differentiation, with acetate and butyrate enhancing FOXP3 expression and regulatory T cell (Treg) expansion while restraining Th17 polarization (Furusawa et al., 2013). Butyrate further increases IL-10 secretion and promotes long-term memory T cell development. These findings highlight the general immunomodulatory properties of SCFAs, underscoring their roles in balancing immune tolerance and inflammation.

Additionally, SCFAs such as butyrate and propionate are HDAC inhibitors and can significantly affect chromatin accessibility and gene transcription in both immune and epithelial cells (Chang et al., 2014). While *Lactobacillus* strains are not obligate anaerobes and thus produce lower levels of butyrate, engineered or co-cultured *Lactobacillus* strains could potentially reach effective local concentrations that activate the HDAC-linked pathway (Wang et al., 2014). Acetate (1–5 mM *in vitro*) has been associated with upregulation of IL-10 and FOXP3 expression, contributing to Treg expansion (Arpaia et al., 2013).

Beyond receptor signaling, SCFAs also influence immune cell metabolism. For example, butyrate promotes oxidative phosphorylation (OXPHOS) in colonic macrophages, triggering an M2-like anti-inflammatory phenotype (Rodríguez et al., 2025). Acetate has been linked to increased antimicrobial peptide production (*e.g.*, RegIIIγ) through NLRP3 activation in IECs (Niu et al., 2023).

It is important to note that the immunological action of SCFAs is both dose- and location-dependent. SCFAs are absorbed at lower concentrations in the systemic circulation compared to the colon. However, studies suggest that SCFAs can activate similar signaling pathways even at micromolar concentrations. Moreover, interactions between Lactobacillus and other gut microbes (*e.g.*, cross-feeding and co-colonization) can amplify the effects of SCFAs, further supporting the idea that immunomodulation mediated by SCFAs is a community-based process (Kaliniak et al., 2024).

SCFAs are well-established immunological modulators that influence host immune responses through multiple mechanisms. Although much of the current knowledge comes from studies on butyrate-producing Clostridia, increasing evidence supports the immunomodulatory roles of SCFAs, including acetate, propionate, and butyrate, and lactate produced by various microbiota, including *Lactobacillus* spp. While lactate is a short-chain carboxylic acid, it is not typically classified as a classic SCFA due to its distinct metabolic roles, such as acting as a cross-feeding substrate for butyrate-producing bacteria. However, lactate shares certain immunomodulatory properties with SCFAs, such as influencing immune responses through specific receptor interactions. Thus, we include lactate here due to its importance in gut microbiology and immunology, where it plays a complementary role alongside SCFAs (Yongsen Wang et al., 2025).

SCFAs can mediate host cell interactions through receptor-mediated signaling and epigenetic mechanisms. The most described receptors for SCFAs are GPR41 (FFAR3), GPR43 (FFAR2), and GPR109A, which are highly expressed on intestinal epithelial cells (IECs), dendritic cells (DCs), neutrophils, and T lymphocytes (Du et al., 2024). SCFAs bind to these G-protein-coupled receptors (GPCRs), triggering anti-inflammatory responses, enhancing mucosal barrier function, and inhibiting the production of pro-inflammatory cytokines such as TNF-α, IL-6, and IL-1β (Ney et al., 2023).

### Influence of *lactobacillus* on gut microbiota and SCFA production: cross-feeding, co-colonization, and cooperative interactions

*Lactobacillus* spp. not only directly contribute to SCFA production but also play a crucial role in modulating the gut microbiota composition. Through interactions with other microbial species, *Lactobacillus* can influence the SCFA pool and contribute to the overall health benefits associated with these metabolites.

One key mechanism by which *Lactobacillus* influences the gut microbiota is cross-feeding, where *Lactobacillus*-produced metabolites, such as acetate and lactate, serve as substrates for other microbes, particularly butyrate-producing bacteria like *Faecalibacterium prausnitzii* and *Anaerobutyricum* spp. (Hatti-Kaul et al., 2018). This cross-feeding enhances SCFA production, particularly butyrate, improving gut barrier function and immune modulation.

Additionally, *Lactobacillus* can co-colonize with other beneficial microbes, fostering a symbiotic relationship that further enriches the gut’s SCFA pool. By promoting the growth of other SCFA-producing bacteria, *Lactobacillus* helps maintain a balanced microbial ecosystem, which supports gut health by promoting beneficial taxa and inhibiting harmful pathogens (Koh et al., 2016).

*Lactobacillus* also influences microbiota diversity, which is critical for maintaining a healthy gut environment. By modulating the growth of different bacterial populations, *Lactobacillus* sustains an optimal balance of SCFA-producing microorganisms, potentially preventing dysbiosis—an imbalance associated with various gastrointestinal and systemic health issues (Markowiak-Kopeć and Śliżewska, 2020). *Lactobacillus* promotes the growth of butyrate-producing bacteria, vital for maintaining gut barrier integrity and modulating inflammation, particularly in conditions like inflammatory bowel disease (IBD) and metabolic syndrome (Fusco et al., 2023).

Recent studies have demonstrated that GPR41 and GPR43 are activated at low concentrations of SCFAs such as acetate and lactate. Specifically, concentrations of 1–5 mM are sufficient to activate these receptors, which are crucial for modulating immune responses and inflammation (Furusawa et al., 2013).

Thus, *Lactobacillus* not only contributes directly to SCFA production but also plays an integral role in shaping the microbial ecosystem that supports SCFA production, ensuring optimal gut health and immune regulation. Understanding these complex interactions is key to designing probiotics that maximize SCFAs' therapeutic potential in clinical applications.

### *Lactobacillus* and Dysbiosis prevention: modulating microbiota for optimal SCFA production

*Lactobacillus* spp. play a key role in maintaining a balanced gut microbiota by preventing dysbiosis, a microbial imbalance that can lead to various gastrointestinal and systemic health issues. Dysbiosis is often associated with reduced microbial diversity and an overgrowth of pathogenic bacteria, which can impair SCFA production and negatively affect gut health.

*Lactobacillus* contributes to the prevention of dysbiosis by promoting a diverse microbial community. By stimulating the growth of beneficial bacteria, *Lactobacillus* helps sustain an optimal balance of SCFA-producing microorganisms. This balanced microbiota is essential for maintaining healthy levels of SCFAs, which are crucial for gut barrier function, immune modulation, and the regulation of metabolic processes (Fusco et al., 2023).

Moreover, *Lactobacillus* exerts antimicrobial effects against pathogenic bacteria by competing for nutrients and producing metabolites, such as lactic acid, that inhibit the growth of harmful microbes. This ability to inhibit pathogenic growth helps prevent microbial imbalances that could otherwise lead to conditions like IBD and metabolic disorders.

By preventing dysbiosis and enhancing the diversity of SCFA-producing bacteria, *Lactobacillus* helps maintain a healthy and resilient gut microbiota, ultimately supporting overall gut health and improving host immune function. Recent studies have shown that SCFAs, particularly acetate and butyrate, play a vital role in modulating immune responses through receptors such as GPR41 and GPR43, which are activated at concentrations as low as 1–5 mM (Furusawa et al., 2013; Zhao et al., 2021). This highlights the therapeutic potential of *Lactobacillus*-based probiotics in preventing and managing gastrointestinal diseases linked to dysbiosis and reduced SCFA production.

### Bioengineering strategies to enhance SCFAs production

Recent advances in synthetic biology and metabolic engineering have enabled the optimization of SCFA biosynthesis in *Lactobacillus* spp., opening opportunities to actualize them as next-generation live biotherapeutic products (LBPs) (Fekete et al., 2023). While the native SCFAs output from *Lactobacillus* is often modest and strain-specific, engineering strategies have demonstrated clear potential to enhance yields, fine-tune metabolite profiles, and improve functionality under gut-like conditions.

One of the most straightforward approaches is the overexpression of native SCFAs-producing genes, such as pta (phosphotransacetylase) and ackA (acetate kinase), to increase flux towards acetate production. Heterologous expression of butyrate biosynthetic genes—such as but (butyryl-CoA: acetate CoA-transferase) and buk (butyrate kinase)—from *Clostridium* species into *Lactobacillus* strains has also been attempted. Although such trials faced challenges like metabolic burden, redox imbalance, and absence of native cofactors, they highlight the feasibility of cross-genus pathway integration.

CRISPR–Cas systems have further expanded engineering capabilities by enabling precise genome editing in LAB, such as promoter replacement, pathway knock-in, and targeted knockouts of competing pathways like ldh (lactate dehydrogenase), thereby redirecting flux from pyruvate towards SCFAs biosynthesis (Goh & Barrangou, 2021; Mu et al., 2022; Zhou et al., 2019). Optimizations of synthetic promoters and ribosome binding sites, such as the use of strong synthetic promoters (*e.g.*, Pgap, Ptac) and engineered ribosome binding sites (RBS), have been reported to improve metabolic control and enhance the production of target metabolites in engineered *Lactobacillus* strains (Zhao et al., 2019; Xie et al., 2024).

Beyond single-strain engineering, co-culture approaches offer a powerful alternative. For instance, co-culturing *Faecalibacterium prausnitzii* with *Bifidobacterium catenulatum* enhanced survival and butyrate production, highlighting a functional cross-feeding interaction (Kim et al., 2020). These co-culture systems, when paired with spatial microencapsulation or quorum-sensing modules, hold promise for developing site-targeted SCFAs delivery strategies.

Engineered strain delivery and survivability are also critical. Encapsulation technologies—such as alginate beads, lipid microgels, and synbiotic formulations—are being leveraged to improve the persistence of SCFA-producing strains and to ensure that engineered metabolic traits are functionally manifested *in vivo* via measurable metabolite output (Nami et al., 2023; Shanuke et al., 2025). These strategies protect strains during gastrointestinal transit, enable targeted colonization, and support sustained SCFAs production.

Furthermore, targeted knockouts such as ldh can divert carbon flux toward acetate or butyrate but often disturb NAD+/NADH homeostasis, leading to reduced growth or altered product spectra. Systems-level modeling (*e.g.*, flux balance analysis) and cofactor regeneration modules are increasingly applied to anticipate and mitigate such trade-offs, ensuring metabolic stability in engineered *Lactobacillus*. Despite these advances, significant challenges remain. The gut environment imposes selective pressures, immune activation, and microbial competition that complicate the performance of engineered strains.

Regulatory and biosafety considerations remain a critical translational bottleneck for genetically modified probiotics. Both the U.S. Food and Drug Administration (FDA) and the European Food Safety Authority (EFSA) have established frameworks that govern the evaluation of live biotherapeutic products. In the U.S., the FDA requires genetically modified *Lactobacillus* strains to undergo Investigational New Drug (IND) applications and compliance with Good Manufacturing Practices (GMP), emphasizing preclinical toxicology, biodistribution, and shedding studies. Similarly, EFSA enforces Qualified Presumption of Safety (QPS) assessments, with additional scrutiny for engineered strains, including requirements for genetic stability, absence of transferable antibiotic resistance markers, and detailed environmental risk assessments.

Notably, case studies such as *Lactococcus lactis* engineered to deliver IL-10 for inflammatory bowel disease illustrate both the promise and the regulatory hurdles. While these strains advanced to safety testing, their clinical progression was slowed by regulatory caution, underscoring the high bar for translational acceptance. In response, biosafety strategies such as auxotrophy, kill-switches, and chassis refactoring are increasingly incorporated to satisfy both regulatory agencies and public acceptance. Similar pathways of oversight and precaution will inevitably apply to engineered *Lactobacillus* strains designed for enhanced SCFAs production.

Addressing these regulatory and biosafety barriers in depth will be indispensable for advancing *Lactobacillus*-based SCFA producers from proof-of-concept to clinically approved therapeutics. In summary, integrating systems biology, synthetic biology, and probiotic science provides unique opportunities to design *Lactobacillus*-based products with improved SCFAs production, paving the way for targeted immunometabolic applications.

### Safety of genetically engineered *lactobacillus* strains

The use of genetically engineered (GE) *Lactobacillus* strains in probiotics raises important concerns regarding their safety for both environmental and human health. While *Lactobacillus* spp. are generally recognized as safe (GRAS) for use in food products, the genetic modification process introduces specific risks that require careful monitoring and regulation.

**Environmental Safety**: To ensure environmental safety, it is critical to prevent the unintended release of GE *Lactobacillus* strains into the environment. Strategies such as containment measures during production and transportation, as well as genetic modifications that allow for controlled degradation of the engineered strains in non-target environments, are essential. For example, the use of genetic circuits that deactivate the strains in the absence of specific environmental triggers is a potential strategy (Zhao et al., 2021).

**Human Health Safety**: For human health, rigorous safety testing is essential before GE *Lactobacillus* strains are released as probiotics. This includes assessing potential pathogenicity, antibiotic resistance, and metabolic changes resulting from genetic modifications. Long-term clinical trials are necessary to evaluate any adverse effects in human populations, particularly those with compromised immune systems. Additionally, monitoring should include:

✓ Antibiotic resistance profiling to ensure GE strains do not acquire or transfer resistance to important antibiotics.
✓ Pathogenicity testing to evaluate the risk of introducing any virulence factors.
✓ Immunological response monitoring in clinical settings to detect any adverse reactions.

competing for nutrients and producing metabolites, such as lactic acid, that inhibit the growth of harmful microbes, reducing the risk of dysbiosis and enhancing gut health. In response to regulatory concerns, biosafety strategies such as auxotrophy, kill-switches, and chassis refactoring are increasingly incorporated to satisfy both regulatory agencies and public acceptance. These strategies are essential for minimizing the risk of unintended environmental or health consequences from the use of GE *Lactobacillus* strains. In conclusion, while genetically engineered *Lactobacillus* strains offer promising probiotic applications, ensuring their safety through these strategies is essential to protect both human health and the environment.

### Therapeutic applications and translational perspectives

The ability of *Lactobacillus*-derived SCFAs to modulate metabolic pathways and host immune responses demonstrates that these beneficial microbes have potential as therapeutic agents. Recent evidence supports the utilization of SCFAs-producing *Lactobacillus* strains for treating inflammatory, metabolic, and neuroimmune diseases in which dysbiosis and disrupted microbial metabolite signaling exist (Koh et al., 2016). Moreover, microbiota-targeted modulation through SCFAs-producing probiotics may have implications for adjunctive therapy in certain infectious diseases (*e.g.*, leptospirosis) where microbiota restoration could support conventional treatment (Petakh et al., 2024).

Low SCFAs levels, particularly butyrate, are strongly associated with impaired barrier function, mucosal inflammation, and dysregulated regulatory T cell responses in inflammatory bowel diseases (IBD) such as Crohn’s disease and ulcerative colitis (Parada Venegas et al., 2019). Although *Lactobacillus* strains are not primary butyrate producers, their acetate and lactate serve as substrates for cross-feeding with butyrogenic commensals, thereby contributing to the restoration of ecological function and mucosal homeostasis (Flint et al., 2024). This cross-feeding interaction is central to the re-establishment of eubiosis, a balanced microbial state in which composition and metabolic outputs—including SCFAs production—are optimized for host health (Petersen and Round, 2014).

Importantly, supplementation with engineered *Lactobacillus* strains or consortium-based formulations has been shown to attenuate colitis severity in preclinical models, likely through enhanced SCFAs output and induction of IL-10 production (Huang et al., 2023). Table 4 summarizes the major SCFAs, their immune-related effects, and their contributions to maintaining eubiosis, providing a framework for linking microbial metabolism to host health.

**Table 4:** Effects of Major SCFAs on Host Health and Eubiosis – Summary of the main immunological, barrier, microbiota, and clinical effects of acetate, butyrate, propionate, and lactate. Reported effects are based on experimental and clinical studies; arrows (↑/↓) indicate an increase or decrease of the respective function.

SCFA	Immune Effects	Barrier Effects	Microbiota Effects	Clinical Implications	References
Acetate	↑ IL-10, ↓ TNF-α	↑ MUC2 secretion	Promotes beneficial taxa	Reduced gut inflammation	(Koh et al., 2016; Louis & Flint, 2017)
Butyrate	↑ Treg cells, ↓ NF-κB activation	↑ Tight junction proteins	Inhibits pathogens	Prevents IBD, enhances gut barrier	(Flint et al., 2024; Parada Venegas et al., 2019)
Propionate	Modulates Th1/Th17 responses	Mild barrier support	Increases microbial diversity	Improves glucose metabolism	(Ashraf and Hassan, 2024; Reichardt et al., 2014)
Lactate	Activates innate immunity	↑ Mucus layer	Supports Bifidobacterium	Benefits infant gut health	(Gänzle, 2015; Ma et al., 2024)

From the perspective of metabolic syndrome, the effects of SCFAs on insulin sensitivity, lipid metabolism, and appetite regulation are predominantly mediated via gut–brain axis signaling, gut and/or endocrine pathways (*e.g.*, GLP-1, PYY) (Ashraf and Hassan, 2024). Acetate - the most common SCFA from *Lactobacillus* - has been shown to reduce hepatic lipogenesis and fat mass in models of diet-induced obesity (Li et al., 2018). Even though robust clinical translation is not yet available, early pilot efforts in humans with *Lactobacillus plantarum* and L. *rhamnosus* strains show some improvements in glycemic markers and systemic inflammation, potentially related to SCFAs-dependent pathways (Ignatiou and Pitsouli, 2024).

Furthermore, neuroimmune and neuropsychiatric disorders, including depression, anxiety, and autism spectrum disorders, have been identified as increasingly relevant to revised gut SCFAs profiles. SCFAs show neuroprotective and anti-inflammatory effects on the central nervous system, mediated through the vagus nerve, HDAC inhibition, and microglial activation (Silva et al., 2020). Recent findings also indicate that SCFAs-mediated modulation of neuroimmune pathways may have therapeutic implications in stress-related disorders, including posttraumatic stress disorder, where SCFAs can influence neuroinflammation and neurotransmitter balance (Petakh et al., 2024).

While these findings are promising, most evidence remains in preclinical models, and translation to human settings faces substantial barriers. Key unresolved issues include dose–response relationships (with few human studies specifying SCFAs concentrations), strain-specific variability in metabolic output, and ecological interactions within the gut microbiome that may affect consistency and safety (Dodero et al., 2024). Moreover, potential unintended consequences—such as competitive suppression of resident butyrogenic bacteria—must be considered before clinical deployment. We propose that moving forward, a precision-microbiome engineering strategy—combining analytical omics, controlled delivery of engineered *Lactobacillus*, and quantitative SCFAs output profiling—offers the most viable pathway to harness the immunometabolic potential of SCFAs in therapeutic contexts safely.

There are many barriers to the advancement of translational applications. These barriers could be individual differences in the microbiome's composition, limited bioavailability of orally administered probiotics, and regulations on genetically modified organisms. In addition, strain selection, formulation stability, and delivery targeted to sites in the gut need to be improved to promote uniformity regarding SCFAs production and therapeutic outcomes (Yupeng Wang et al., 2025). Nevertheless, applying *Lactobacillus*-derived SCFAs for therapeutic outcomes using precision microbiome-mediated approaches is a viable pathway forward. With the maturation of synthetic biology tools and improved high-resolution omics profiling, the rational use of SCFAs-targeted probiotics is expected to be an important part of next-generation gut–immune–related therapeutics.

### Clinical and translational insights into SCFAs-Producing *lactobacillus* strains

While much of the research on *Lactobacillus*-derived SCFAs remains preclinical, a growing body of literature moves the field toward clinical applicability. Several new trials and observational studies indicate that some strains of *Lactobacillus* may offer SCFA-mediated health benefits for metabolic, gastrointestinal, and neuroimmune disorders. For example, a double-masked, placebo-controlled supplementation study with *Lacticaseibacillus paracasei* F19 in obese individuals showed improved insulin sensitivity and small increases in serum acetate concentrations linked to G protein-coupled receptor 43 (GPR43) activation and secretion of GLP-1 (Chambers et al., 2015). In patients with irritable bowel syndrome (IBS), L. *plantarum* 299v has been associated with reduced intestinal permeability and systemic inflammation, potentially due to the SCFA-dependent upregulation of tight junction proteins. A randomized trial where children with atopic dermatitis received L. *rhamnosus* GG showed increased fecal acetate concentrations and increased IL-10 expression in their peripheral blood mononuclear cells, indicating immune system modulation (Wickens et al., 2012). Notably, immunological effects such as IL-10 upregulation are typically observed *in vitro* at SCFAs concentrations between 0.1–10 mM (Smith et al., 2013); however, the extent to which *Lactobacillus*-derived SCFAs can reach these levels in the human gut remains to be established (M. Vinolo et al., 2011). However, these clinical studies vary considerably in design and sample size—most are small-scale, short-term, and heterogeneous in dietary or environmental confounders—making it difficult to draw firm causal inferences. Critical evaluation of trial quality and replication across larger, more diverse cohorts is required to substantiate the therapeutic role of *Lactobacillus*-derived SCFAs.

Nevertheless, attributing mechanistic effects directly to SCFAs in human studies is difficult because of inconsistencies in absorption, host metabolism, SCFAs measurement, and microbiota composition. Additionally, most of the current trials do not screen outcomes by SCFAs profile (personalized based on genetic composition or microbiota profile), nor do they use functional biomarkers such as engagement with receptors on the host (*e.g.*, GPR43 expression) or epigenetic readouts (*e.g.*, HDAC activity). Future clinical trials to maximize translational efficacy will also require standardization of SCFAs quantification methodologies (such as GC–MS profiling), microbiome-wide association studies (MWAS) to identify responders, and host transcriptomic/immunologic correlates of SCFAs activity, and using engineered or consortial strains with confirmed outputs of SCFAs. This represents a potential opportunity to validate causal mechanisms further. From a perspective of optimizing dosing and application of *Lactobacillus* in therapeutic contexts - moving from nutritional essences to true microbiome therapeutics, integrated studies investigating combinations and interactions between *Lactobacillus* and SCFAs will be conducted to facilitate an understanding of these facts, justify why and how to use doses, and hopefully expand mechanism characterization.

Moving forward, the clinical translation of *Lactobacillus*-derived SCFAs will require a multi-tiered framework that combines rigorous safety evaluation, standardized SCFAs quantification, and precision-based patient stratification. Trials should incorporate dose–response designs and functional biomarkers (*e.g.*, SCFAs receptor engagement, HDAC activity) to directly link microbial outputs with host responses — mechanisms supported by human and preclinical research (Du et al., 2024). Moreover, integrated multi-omics readouts, including metabolomics, immunoprofiling, and host transcriptomics, will be essential to delineate causal pathways (Du et al., 2024). Regulatory and translational success will also depend on the development of engineered or consortial *Lactobacillus* strains with validated SCFAs production capacity and stable delivery platforms — proof-of-concept studies are encouraging (Dempsey & Corr, 2022; Thananimit et al., 2022). Collectively, this framework emphasizes moving beyond associative data toward reproducible, mechanism-based clinical outcomes.

### Challenges and future directions

Although interest in SCFAs-generating *Lactobacillus* strains as therapeutic agents is growing, many fundamental scientific, technical, and translational challenges remain to be resolved. These gaps must be addressed to advance from theoretical and proof-of-concept studies toward clinically relevant and regulatory-approved applications.

### Strain-specificity and variable SCFAs production

SCFAs production in *Lactobacillus* is highly strain-specific and relies on genetic background, metabolic potential, and ecological environment. *Lactobacillus* species can show significant differences in metabolite yields, protein expression (including enzymes), and abilities to utilize dietary substrates, even in the same species (Papadimitriou et al., 2015). This specificity restricts the extent to which findings can be translated to other *Lactobacillus* strains. It underscores the need to validate SCFAs-producing *Lactobacillus* strains on a strain-by-strain basis under physiologically relevant conditions.

### Context-dependent immunological responses

SCFAs have been shown to have immunoregulatory activity, but their immunological outcomes are context-dependent on dosage, site of action, and host immunity status. For example, acetate can be anti-inflammatory in the colon but pro-inflammatory in the case of a systemic infection (Yap et al., 2021). Future lines of inquiry must employ controlled gnotobiotic approaches, a multi-omics approach, and cell-type-specific assessments to tease out more mechanistic links.

### Engineering complexity and safety issues

Genetically modified *Lactobacillus* for increased SCFAs production poses a challenge because of biosafety concerns, the engineered strain's stability maintenance, and regulatory considerations. Engineered strains should ideally retain their probiotic properties, including the ability to resist horizontal gene transfer, and be replete with biocontainment modules to ensure safety in case of accidental release (Moon, 2024). Additionally, engineered synthetic constructs are often burdensome to the engineered strain, decreasing *in vivo* strain fitness.

### Efficacy of delivery and colonization

Getting SCFAs reliably delivered to the distal gut represents a technical/biological barrier. Most *Lactobacillus* strains, including engineered strains, can only transiently colonize the gut, and the rate of SCFAs production may be affected by oxygen exposure, bile salts, and available nutrient competition (Zeise et al., 2021). Promising technology, such as encapsulation strategies paired with a well-defined prebiotic oligosaccharide and/or engineered, defined synthetic consortia, is undoubtedly a step in the right direction. However, it remains a work in progress requiring refinement.

### Absence of standardization and translational pipelines

The field lacks standardized assays that quantify SCFAs production, receptor engagement, or immune outcomes across models. In addition, few longitudinal clinical trials examine SCFAs-producing probiotics in well-defined patient populations. Greater collaboration between bioengineers, microbiologists, immunologists, and clinicians is necessary to fill these gaps. Going forward, our research agenda needs to emphasize:

✓ Mapping strain-function relationships using high-throughput screening and machine learning;
✓ developing modular genetic toolkits for sustained SCFAs production;
✓ creating dose–response conventions for host–SCFAs interactions; and
✓ engineering rationally-designed microbial consortia to achieve SCFAs on par with or over a natural SCFAs-producing ecosystem.

Ultimately, to realize the full therapeutic potential of *Lactobacillus*-derived SCFAs, a systems-level, multidisciplinary approach is necessary. One that incorporates synthetic biology, immunology, ecological modeling, and clinical science to develop precision microbiome interventions for human health.

## Challenges and limitations

**Strain variability**: Variability in SCFA production between different *Lactobacillus* strains poses a major challenge for optimizing therapeutic and functional applications. Strain-specific differences in metabolic pathways and gene expression result in inconsistent SCFA yields, which can limit the reproducibility of results across studies.

**Context-dependency**: The effects of SCFAs are highly dependent on environmental and physiological conditions. Variations in diet, microbiota composition, and the presence of other metabolites can influence the outcomes of SCFA-based interventions, complicating their application in diverse clinical contexts.

**Engineering complexity**: Metabolic engineering of *Lactobacillus* to enhance SCFA production faces challenges related to metabolic burden, cofactor availability, and the stability of genetically modified strains. These hurdles must be addressed to ensure the scalability and effectiveness of engineered probiotics for clinical use.

## Conclusion

In conclusion, *Lactobacillus*-derived SCFAs represent promising therapeutic agents due to their ability to modulate immune responses and support gut health. Despite the challenges of strain variability, context-dependency, and engineering complexity, advances in synthetic biology and metabolic engineering offer potential solutions for optimizing SCFA production. Future research should focus on enhancing SCFA yields, understanding strain-specific effects, and developing targeted probiotic therapies. By overcoming these hurdles, *Lactobacillus*-based SCFAs can be harnessed as effective tools in treating inflammatory, metabolic, and neuroimmune diseases. The integration of multi-omics approaches and precision microbiome engineering will be critical for translating these concepts into clinically viable applications.

## Funding

Not applicable.

## Clinical trial number

Not applicable.

## Declarations

### Ethical approval

Not applicable.

### Availability of data and materials

The datasets used and/or analyzed during the current study are available from the corresponding author on reasonable request.

## CRediT authorship contribution statement

**Yousef Nami:** Project administration, Supervision, Validation, Writing – original draft. **Milad Shaghaghi Ranjbar:** Writing – original draft, Data curation. **Mahmoudreza Modarres Aval:** Writing – original draft, Data curation. **Babak Haghshenas:** Project administration, Supervision, Validation, Writing – original draft.

## Declaration of competing interest

The authors declare that they have no competing interests.
